# Low-Cost Laser Micromachining Super Hydrophilic–Super Hydrophobic Microgrooves for Robotic Capillary Micromanipulation of Microfibers

**DOI:** 10.3390/mi12080854

**Published:** 2021-07-21

**Authors:** Bo Chang, Yuhang Feng, Jialong Jin, Quan Zhou

**Affiliations:** 1 College of Mechanical and Electrical Engineering, Shaanxi University of Science and Technology, Xi’an 710021, China; 1905003@sust.edu.cn (Y.F.); 1905004@sust.edu.cn (J.J.); 2 School of Electrical Engineering, Aalto University, FI-00076 Aalto, Finland; quan.zhou@aalto.fi

**Keywords:** laser micromachining, capillary self-alignment, super hydrophilic–super hydrophobic patterned surfaces, microfibers, microgrooves

## Abstract

Capillary self-alignment technique can achieve highly accurate and fast alignment of micro components. Capillary self-alignment technique relies on the confinement of liquid droplets at receptor sites where hydrophobic–hydrophilic patterns are widely used. This paper reports a low-cost microsecond pulse laser micromachining method for fabrication of super hydrophilic–super hydrophobic grooves as receptor sites for capillary self-alignment of microfibers. We investigated the influence of major manufacturing parameters on groove sizes and wetting properties. The effects of the width (20 µm–100 µm) and depth (8 µm–36 µm) of the groove on the volume of water droplet contained inside the groove were also investigated. We show that by altering scanning speed, using a de-focused laser beam, we can modify the wetting properties of the microgrooves from 10° to 120° in terms of the contact angle. We demonstrated that different types of microfibers including natural and artificial microfibers can self-align to the size matching super hydrophilic–super hydrophobic microgrooves. The results show that super hydrophilic–super hydrophobic microgrooves have great potential in microfiber micromanipulation applications such as natural microfiber categorization, fiber-based microsensor construction, and fiber-enforced material development.

## 1. Introduction

Capillary self-alignment technique can achieve highly accurate and fast alignment of micro components [1,2,3,4]. Minimization of surface energy is the fundamental principle behind capillary self-alignment, where the gradient of the surface energy drives the micro components towards target receptors [5,6,7]. Capillary self-alignment relies on the liquid droplet confinement inside the receptor, where the liquid droplet takes the shape of the receptor. The receptor should have desired wetting or topological properties in contrast to that of the background. The droplet confinement has been demonstrated using a hydrophilic receptor on hydrophobic background [8], on superhydrophobic background [9,10], an oleophilic receptor on oleophobic background [11], and topological protruding receptor [12,13,14]. These receptor sites are usually fabricated using lithography technology, which can reach high manufacturing accuracy but is often costly and requires a cleanroom environment. Another method for fabricating receptor sites is laser micromachining technique, which has been used to fabricate microgrooves for construction of micro devices such as micro pumps [15], micro mixers [16], micro reactors [17], and micro fuel cells [18]. We previously reported that laser micromachining technology can be used to fabricate grooved surfaces for capillary self-alignment of microchips [19].

In this paper, we study the influences of fabrication parameters of the grooved surfaces on capillary self-alignment of microfibers, and we show that by altering scanning speed using a de-focused laser beam, we can modify the wetting properties of the microgrooves from 10° to 120° in terms of the contact angle. We design and fabricate micro grooves with different sizes and wetting properties of shapes matching the microfibers. We investigate the key fabrication parameters including the number of scanned lines, number of scans, scanning speed, and their effects on the geometry and wetting properties of the microgrooves. We also study the influences of the resulted width and depth of the groove on the volume of water droplet confined inside the grooves. Finally, we demonstrate that diverse types of microfibers, including natural and artificial microfibers, can self-align to super hydrophilic–superhydrophobic microgrooves.

## 2. Materials and Methods

### 2.1. Laser Micromachining of Super Hydrophilic–Super Hydrophobic Grooves

**Figure 1:**
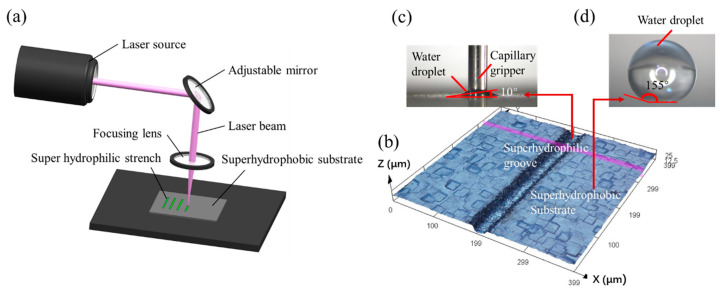
Schematic of laser micromachining of super hydrophilic–super hydrophobic grooves and fabricated grooves: (**a**) schematic of laser micromachining of super hydrophilic–super hydrophobic grooves; (**b**) 3D construction of fabricated grooves; (**c**) water contact inside the groove; (**d**) water contact angle outside the groove.

In this paper, we use a low-cost microsecond pulse laser micromachining method to fabricate super hydrophilic–super hydrophobic grooves for capillary self-alignment of microfibers. The grooves are fabricated on silicon substrates. First, a super hydrophobic coating (WHOLE-NANO, SPN-62) is sprayed on a silicon substrate and dried at room temperature for 24 h. The substrate is then patterned with a UV laser marking machine HGTECH LU-5 (Huagong Ltd., Wuhan, China) with a 5 W power and a 355 nm wavelength. Figure 1a shows the schematic of laser marking process. Laser marking machine uses a focused beam of laser to mark the surface of a material. When the light beam interacts with the material surface, it alters the properties of the surface and forms designed patterns. To fabricate super hydrophilic–super hydrophobic grooves, the laser marking machine is set to a pulse duration of 1 µs, a current of 1 A, a scanning speed of 1500–2000 mm/s, and a frequency of 100 kHz. By varying the number of scanned lines (the number of parallel scanning lines of the laser spot) and the number of scans, different widths and depths of the grooves were fabricated. The fabricated grooves have widths of 20 µm to 100 µm and depths of 8 µm to 36 µm. By adjusting the scanning speed, grooves with water contact angles ranging from 10° to 120° can be fabricated. Figure 1b shows one example of fabricated grooves with a width of 20 µm, a depth of 8 µm, a water contact angle of 10° inside the fabricated groove (Figure 1c), and a water contact angle of 155° outside the groove (Figure 1d).

### 2.2. Robotic System for Capillary Self-Alignment of Microfibers

**Figure 2:**
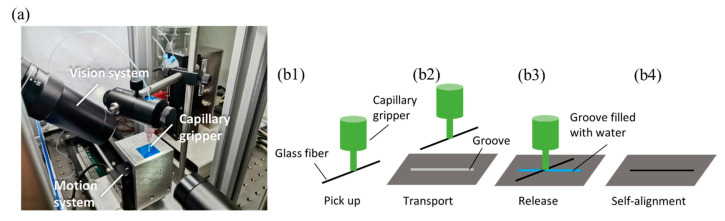
Robotic system and schematic of capillary self-alignment of microfiber: (**a**) robotic manipulation system for capillary self-alignment; (**b1**–**b4**) schematic of capillary self-alignment.

The robotic manipulation system for performing capillary self-alignment test is shown in Figure 2a, and it was adapted from a prior study [20]. The system consists of a capillary gripper, a 3D motion system, and a vision system. The capillary gripper can produce droplets and pick-and-place the microfiber; the motion system moves the fabricated grooves in three dimensions; and the vision system monitors the capillary self-alignment process and provides visual feedback. The schematic of the capillary self-alignment is shown in Figure 2b1–b4. Initially, a microfiber is picked up by a capillary gripper, and then the fiber is transported to a substrate with microgrooves; the water droplets quickly spread in the groove, and droplets are confined inside the groove due to the groove’s super hydrophilicity and the substrate’s super hydrophobicity; this is while the fiber aligns itself to the fully wetted groove; finally, the water droplets evaporate, leaving the fiber aligned to the groove.

## 3. Results

### 3.1. Criterion for Droplet Confinement

**Figure 3:**
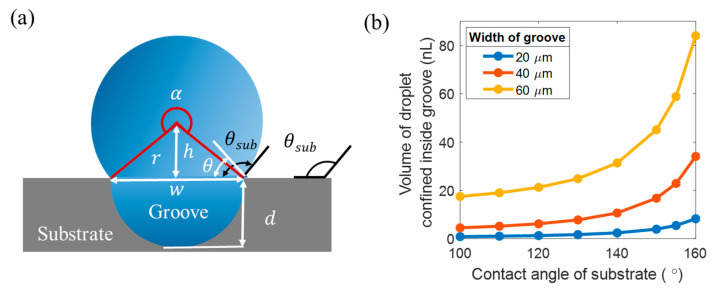
Droplet confinement inside groove according to Gibbs’ criterion. (**a**) Liquid droplet can be confined inside the groove until the apparent contact angle θ reaches $\theta_{sub}$; (**b**) the volume of the droplet confined inside the groove with different widths increases as the contact angle of the substrate increases.

Capillary self-alignment relies on the droplet confinement inside the grooves. There are two ways to confine the liquid droplet inside the groove. One is based on the large wetting contrast for planar patterns, and the other method utilizes sharp edges to pin the liquid according to Gibbs’ criterion [21]. In our case, the droplet confinement is achieved by both sharp edge and wetting contrast. The amount of liquid that can be confined inside the groove is estimated based on Gibbs’ criterion, as shown in Figure 3; the apparent contact angle of a liquid droplet inside the groove θ may extend over a range of angles. This indicates that the liquid droplet with increased volume can be confined inside the groove until the apparent contact angle θ reaches $\theta_{sub}$. As the volume of the liquid continuously increases, and the contact angle θ exceeds $\theta_{sub}$, the droplet contact line crosses the edge of the groove and the liquid droplet spreads on the substrate. The maximum volume of the droplet confined inside the groove can be calculated by:

(1) $$V=\frac{1}{2}\pi {\left(\frac{w}{2}\right)}^{2}l+Al$$

where l, w, are the length and the width of the groove, and A is the area of the circular segment above the groove, which can be calculated by:

(2) $$A=\frac{\alpha \pi r^{2}}{360}+\frac{wh}{2}$$

where r is the radius of the circular segment, α is the central angle in degrees and $\alpha =2\theta_{sub}$, h is the height of the triangular portion and $h=r\sin\left(\theta_{sub}-90{}^{\circ}\right)$. Given the apparent contact angle of the liquid droplet on the superhydrophobic substrate $\theta_{sub}$ is 155°, the length of the groove l is 3 mm, the depth of the groove d is 8 µm, the maximum volumes of the droplet confined inside a 20 µm, 40 µm, and 60 µm wide groove are around 5.7 nL, 23 nL, and 51 nL, respectively, which are estimated using Equation (1). Figure 3b shows the relationship between the contact angle of the substrate and the volume of the droplet confined inside the groove with different widths. The simulation shows that the volume of the droplet confined inside the groove increases as the contact angle of the substrate increases. Therefore, to confine a large amount of droplet inside the groove and meet the requirement of the surface of the water inside the groove higher than the top surface of the groove, the superhydrophobic substrate is preferred.

### 3.2. Influence of Manufacturing Parameters on Size of the Grooves

**Figure 4:**
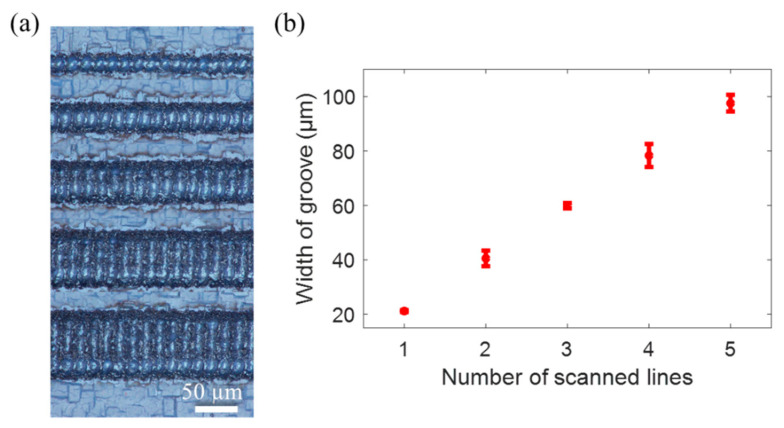
Influence of the scanned lines on the width of the groove. (**a**) Fabricated grooves with widths of 20 µm, 40 µm, 60 µm, 80 µm, and 100 µm; (**b**) relationship between the number of the scanned lines and the width of the groove.

The size of the grooves plays an important role in capillary self-alignment of microfibers. A series of experiments were conducted to investigate the key manufacturing parameters and their effect on groove size. The grooves with different width were fabricated by adjusting the number of scanned lines. A single scanned line has a width of about 20 µm. Parallel lines were machined to create grooves of varying widths. The spacing between parallel scanned lines is set to 20 µm. Figure 4a shows the fabricated grooves with widths of 20 µm, 40 µm, 60 µm, 80 µm, and 100 µm. The relationship between the number of scanned lines and the width of the groove (Figure 4b) shows that as the number of scanned lines increases, so does the width of the groove. 

**Figure 5:**
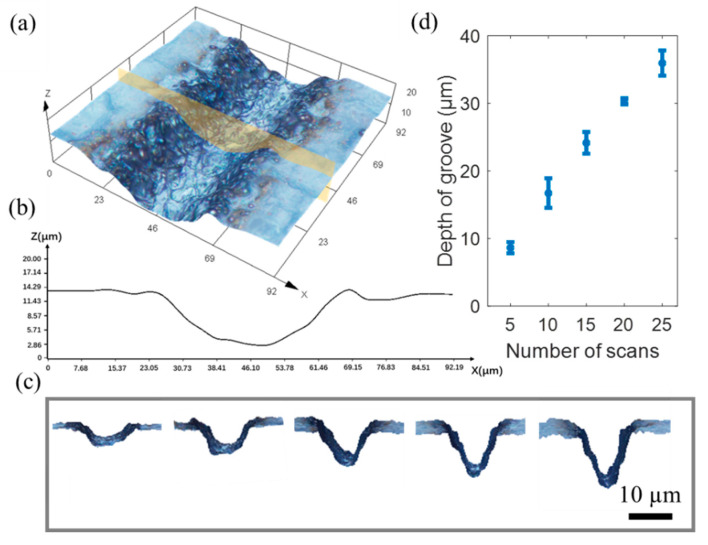
Influence of the number of scans on the depth of the groove. (**a**) 3D construction of fabricated groove; (**b**) profile of fabricated groove; (**c**) cross section of fabricated grooves with different depths; (**d**) relationship between the number of the scans and the depth of the groove.

The depth of the grooves is determined primarily by the number of scans. To fabricate the groove with a different depth, by repeatedly scanning over the same location, the depth of the groove can be adjusted. Figure 5a shows the 3D reconstruction of a fabricated groove with a width of 40 μm and a depth of 9.8 µm, as shown in the profile (Figure 5b). Figure 5c depicts a cross section of the groove with various depths. Figure 5d shows that the number of scans is proportional to the depth of the grooves.

### 3.3. Fabrication of Grooves with Different Wetting Properties

**Figure 6:**
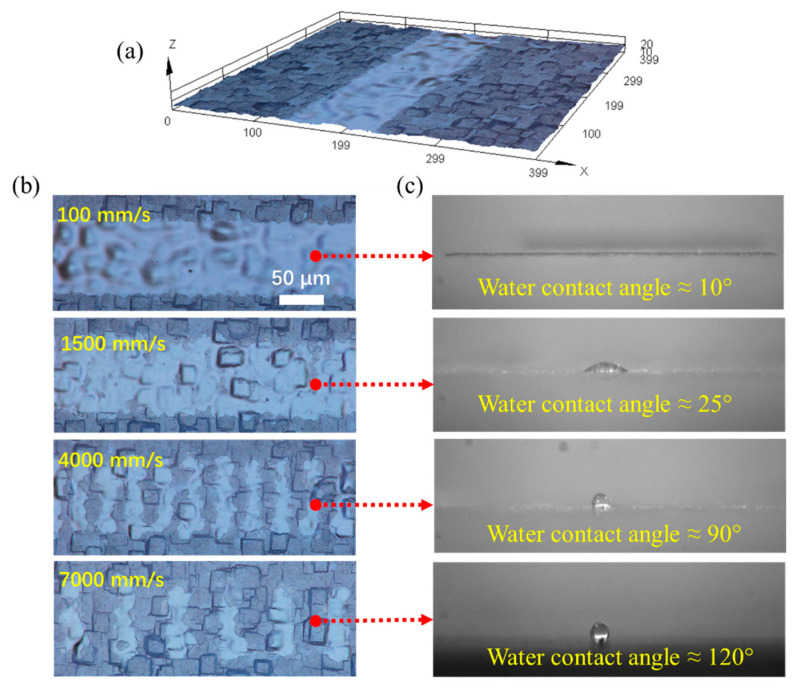
Fabricated grooves with different wetting properties. (**a**) A 3D reconstruction of a fabricated groove; (**b**) 2D images of grooves fabricated at scanning speeds of 100 mm/s, 1500 mm/s, 4000 mm/s, and 7000 mm/s; the dark areas have superhydrophobic coating, the superhydrophobic coating is removed in the light areas; (**c**) measured water contact angles of 10°, 25°, 90°, and 120° on groove structures.

To fabricate grooves with varying wetting properties, we first set the laser out of focus, reducing the intensity of the laser beam significantly compared with a focused laser beam. Then, we adjusted the scanning speed to change the morphology of the surfaces. The groove structures fabricated at different scanning speeds of 100 mm/s, 1500 mm/s, 4000 mm/s, and 7000 mm/s are shown in Figure 6.

Given the pulse frequency of the laser beam Frequency=100 kHz, and the scanning speed of the laser beam Scan_Speed=100, 1500, 4000, 7000 mm/s, the spacing between two pulses/light areas can be estimated as Frequency/Scan_Speed. Therefore, the estimated spacing of two pulses is approximately 1 µm, 15 µm, 40 µm, and 70 µm at scanning speeds of 100 mm/s, 1500 mm/s, 4000 mm/s, and 7000 mm/s, respectively. It can be seen clearly in Figure 6b that the spacing between two pulses/light areas is about the same as the estimated value, which is 40 µm and 70 µm in the case of scanning speeds of 4000 mm/s and 7000 mm/s. When the scanning speed is very low, such as 100 mm/s, the spacing is only about 1 µm; therefore, the removed areas are highly overlapped. Furthermore, Figure 6b shows that the area removed per pulse (light areas in 4000 mm/s and 7000 mm/s cases) is about 30 µm wide, which mainly depends on the laser beam pulse duration; in our case, it is 1 µs. Figure 6c depicts images of water contact angles on fabricated grooves. When the scanning speed was set to 100 mm/s, the entire superhydrophobic coating and part of the silicon layer were removed, leading to groove structures that are super hydrophilic. As the scanning speed increases, the number of pulses per length decreases, and clear spacings are observed for the cases of 4000 mm/s and 7000 mm/s. Therefore, the superhydrophobic coating was removed only partially or interlaced on the scanned area, leading to different wetting properties. The wetting properties were tested with a non-contact dispenser (model: Gesim/PicPIP) that can dispense water droplets in the groove. When the water contact angle of the groove is 10°, the droplets completely wet the groove. Only a small portion of the groove was wetted as the water contact angle of the groove increased to 25°. The droplets do not spread in the groove when the groove is hydrophobic. Table 1 displays the measured water contact angle in the groove as a function of scanning speed. The results show that it is preferable for the groove to be super hydrophilic, so that the groove can be completely wetted by water droplets for microfiber capillary self-alignment.

### 3.4. Influence of Size of Groove on Droplet Confinement

**Figure 7:**
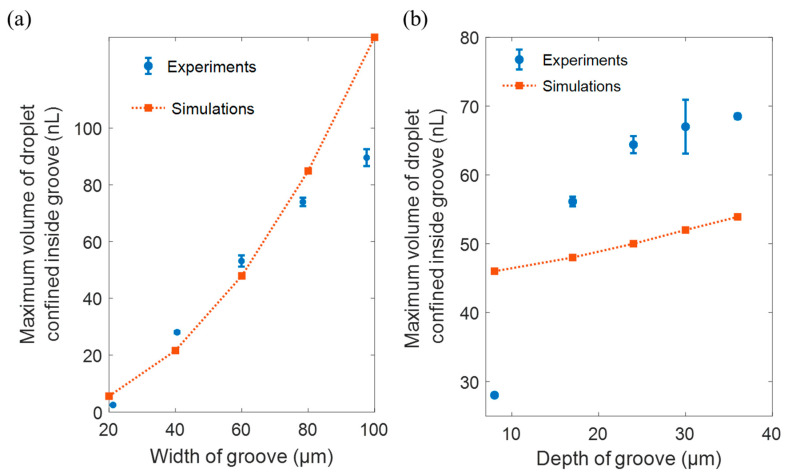
Influence of the width and depth of the groove on maximum volume of droplet confined inside the groove: (**a**) volume of droplet confined inside the groove as the function of the width of the groove; (**b**) volume of droplet confined inside the groove as the function of the depth of the groove.

Droplet confinement is key for capillary self-alignment; we experimentally studied the influence of the size of the groove on the droplet confinement. We tested the grooves of sizes ranging from 20 µm to 100 µm in width, 8 µm to 36 µm in depth, all of which were 3 mm long. The water contact angles in the groove and on the substrate are about 10° and 155°, as mentioned earlier. The volume of the droplet confined inside the groove is defined as the maximum volume of the droplet before spreading out. A series of droplets was dispensed at the groove, at a rate of one droplet per 25 milliseconds, with the volume of 92 pl per droplet (calibrated based on the specifications provided by the manufacturer of the non-contact dispenser) until the droplet spreads out from the groove to the substrate. We investigated both the relationship between the maximum confined volume as the function of the width and the depth. The depth of the groove was fixed at 8 µm for tests of different widths, and the width of the groove was fixed at 40 µm for the tests of different depths. Each test was repeated 5 times. Figure 7 shows the influence of the width and the depth of the groove on the volume of the droplet confined inside the groove. The *x*-axis represents the width and the depth of the groove, and the *y*-axis represents the maximum volume of the droplet confined inside the groove, which consists of a mean of 5 repetitions with the standard derivation. The orange squares connected with dotted lines in Figure 7a,b represent the maximum volume of the liquid droplets confined inside the groove estimated based on Equation (1). The blue bars represent the experimental results of the confined volume. For the influence of the groove width, the experimental results reveal that the relationship between width and volume is mainly linear. The theoretical prediction for droplet volume confined inside a 20 µm, 40 µm, and 60 µm wide groove with a depth of roughly 8 m is about 3 nL, 28 nL, and 53 nL, which is close to the estimation based on Gibbs criterion (4.3 nL, 26 nL, 55 nL). The discrepancies between experimental and theoretical prediction increase at 80 and 100 µm, which we attribute to the jagged edges of the groove. For the influence of the depth of groove, the experimental data show that the relation is positive and saturating when the depth increases. The theoretical model predicted only a positive linear relationship, even though the trend is the same, the volume and gradient are largely different. We attribute this discrepancy to the combined influence of the jagged edges and irregular topography caused by the heating effect of laser manufacturing process. Nevertheless, both experimental results and theoretical estimations show that the maximum volume of the droplet confined inside the groove increases as the width and the depth of the groove increase. It is suggested that the volume of the droplet for self-alignment should not exceed the maximum volume of the droplet confined inside the groove to achieve the successful capillary self-alignment of microfibers.

### 3.5. Demonstration of Capillary Self-Alignment of Microfibers on Fabricated Microgrooves

**Figure 8:**
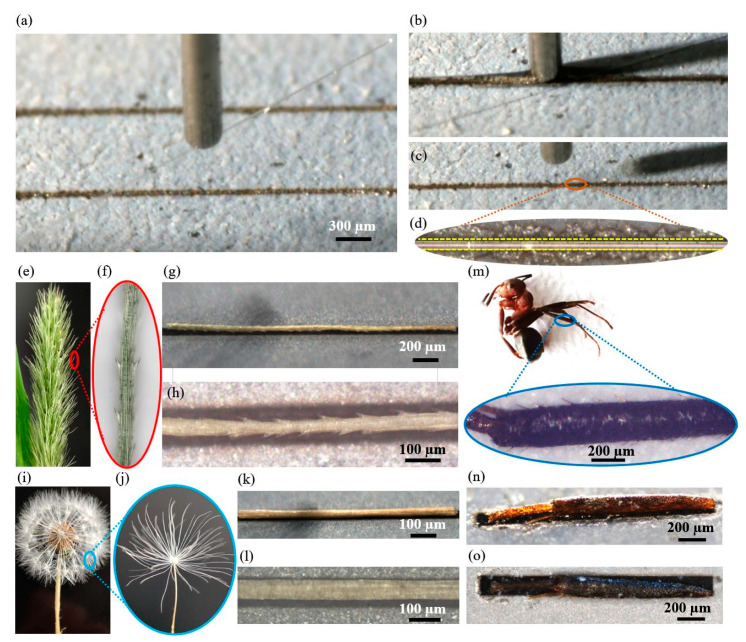
Demonstration of capillary self-alignment of different types of microfibers on shape matching super hydrophilic–super hydrophobic grooves: (**a**) A glass microfiber with a diameter of 15 µm is picked up and transported to a substrate with grooves by a capillary gripper; (**b**) the fiber is released onto the groove and the liquid fully wets the super hydrophilic groove; (**c**) the glass fiber is self-aligned to the groove and water droplets evaporate; (**d**) a zoomed image of a glass microfiber aligned to the groove; (**e**) microscopic image of a dog tail grass; (**f**) a zoomed image of one tip of a dog tail grass; (**g**) titled view of an aligned dog tail grass in the groove; (**h**) top view of an aligned dog tail grass in the groove; (**i**) microscopic image of a dandelion seed; (**j**) zoomed image of a tip of dandelion seed; (**k**) titled view of an aligned dandelion seed in the groove; (**l**) top view of an aligned dandelion seed in the groove; (**m**) microscopic image of an ant; (**n**) zoomed image of a leg of the ant; (**n**) titled view of an aligned ant leg in the groove; (**o**) top view of an aligned ant leg in the groove.

To demonstrate that the fabricated hydrophilic–super hydrophobic grooves can be used to align microfibers, we carried out capillary self-alignment tests using various types of microfibers including both natural and artificial fibers, e.g., dog tail grass, dandelion seeds, legs of ants, and glass fibers. The microfibers have diameters ranging from 15 µm to 200 μm and the length of around 3 mm. The fabricated super hydrophilic–super hydrophobic microgrooves match the shape of the microfibers, having the width of 20–220 μm, depth of 24 μm, length of 3 mm, and the water contact angle of 10°. Figure 8 shows a demonstration of the capillary alignment of a glass microfiber with a diameter of 15 µm on the super hydrophilic–super hydrophobic groove. Firstly, a glass fiber is picked up and transported to a groove by a capillary gripper (Figure 8a); next, the fiber is released onto the groove and the liquid droplet fully wets the super hydrophilic groove (Figure 8b); then, the fiber is self-aligned to the groove, and the water droplets evaporate (Figure 8c). A zoom image of an aligned microfiber inside the groove is shown in Figure 8d, where the yellow dashed lines represent the edges of the groove, and the results show that the self-alignment of microfiber can be achieved. Figure 8e–h shows a dog tail grass self-aligned to a shape matching microgroove. It appears that the self-alignment is not hampered even though the dog tail grass has little spikes (Figure 8h). Capillary self-alignment has also been proven with a dandelion seed, as shown in Figure 8i–k and leg of an ant (Figure 8m–o). It is worth noting that both the dandelion seed and the leg of an ant are slightly bent, but this does not appear to impair the capillary self-alignment process.

## 4. Conclusions

This paper reports a simple and low-cost laser micromachining method for fabrication of super hydrophilic–super hydrophobic grooves for capillary self-alignment of microfibers. We investigated key manufacturing parameters and its effect on the sizes and wetting properties of the grooves. We studied the influences of the width (20 µm–100 µm) and the depth (8 µm–36 µm) on the volume of water droplet confined inside the groove. The results reveal that the groove’s width and depth are proportional to the number of scanned lines and scans, respectively. We further demonstrated that, by adjusting the scanning speed of a de-focused laser beam, we can change the microgrooves’ wetting properties from 10° to 120° in terms of the contact angle. We demonstrated that diverse types of microfibers, including both natural and artificial microfibers, can self-align to super hydrophilic–superhydrophobic microgrooves. The results suggest that super hydrophilic–super hydrophobic microgrooves have a lot of potential in microfiber micromanipulation applications such as natural microfiber categorization, fiber-based microsensor construction, and fiber-enforced material development.

## Figures and Tables

**Table 1:** Influence of scanning speed on wetting property.

Scanning Speed (mm/s)	Water Contact Angle
100	10°
1500	25°
4000	90°
7000	120°
